# Clinical evaluation of a novel subcutaneous lactate monitor

**DOI:** 10.1007/s10877-021-00685-1

**Published:** 2021-04-10

**Authors:** Nitsan Dror, John Weidling, Sean White, Francesca Ortenzio, Samir Shreim, Mark T. Keating, Hoang Pham, Shlomit Radom-Aizik, Elliot Botvinick

**Affiliations:** 1 Pediatric Exercise and Genomics Research Center, Department of Pediatrics, School of Medicine, University of California Irvine, Irvine, USA; 2 Pediatric Department, Meir Medical Center, Child Health and Sports Center, Sackler School of Medicine, Tel Aviv University, Tel Aviv, Israel; 3 Beckman Laser Institute and Medical Clinic, University of California Irvine, Irvine, USA; 4 Department of Dermatology, University of California Irvine Health, Irvine, USA; 5 Edwards Lifesciences Center for Advanced Cardiovascular Technology, University of California Irvine, Irvine, USA

**Keywords:** Continuous lactate monitor, Lactate, Lactic acid, Real-time monitoring, Exercise physiology, Optode

## Abstract

Lactate levels are commonly used as an indirect measure to assess metabolic stress in clinical conditions like sepsis. Dynamic lactate measurements are recommended to assess and guide treatment in patients with shock and other critical care conditions. A minimally invasive, continuous lactate monitor has potential to improve clinical decisions and patient care. The purpose of the study was to evaluate continuous lactate measurements of a novel enzymatic Continuous Lactate Monitor (CLM) developed in our laboratory. Lactate levels were monitored during incremental cycling exercise challenges as a tool for hyperlactatemia. Six healthy individuals 18–45 y/o (4 males, 2 females) participated in the study. CLM devices were inserted subcutaneously in the postero-lateral trunk below the renal angle, one hour before the exercise challenge. Each exercise challenge consisted of a 3 to 12-min warm up period, followed by up to 7, 4-min incremental workload bouts separated by rest intervals. Continuous lactate measurements obtained from CLM were compared with commercial lactate analyzer (Abbott iSTAT) measurements of venous blood (plasma) drawn from the antecubital vein. Blood was drawn at up to 25 time points spanning the duration of before exercise, during exercise, and up to 120 min post exercise. Area under the curve (AUC), and delay time were calculated to compare the CLM readings with plasma lactate concentration. Average plasma lactate concentration increased from 1.02 to 16.21 mM. Ratio of AUC derived from CLM to plasma lactate was 1.025 (0.990–1.058). Average dynamic delay time of CLM to venous plasma lactate was 5.22 min (2.87–10.35). Insertion sites examined 48 h after CLM removal did not show signs of side effects and none required medical attention upon examination. The newly developed CLM has shown to be a promising tool to continuously measure lactate concentration in a minimally invasive fashion. Results indicate the CLM can provide needed trends in lactate over time. Such a device may be used in the future to improve treatment in clinical conditions such as sepsis.

## Introduction

L-Lactate (subsequently referred to as lactate) is a product of the glycolysis pathway via pyruvate and has been used as a biomarker of oxygen deficits in tissues during events such as hypovolemic, septic, or cardiogenic shock [1, 2]. Moreover, lactate concentration is used as one criterion for septic shock diagnosis [3] and studies have shown that delays in lactate measurement are associated with delayed antibiotic treatment and increased mortality in patients with initial intermediate or elevated lactate levels [4]. In contrast, lactate-guided therapy in the intensive care unit (ICU) significantly reduced ICU length of stay and mortality in patients [5]. Notably, a case presentation by Bakker et al. emphasizes how a single measurement of venous lactate can provide the clinician better risk assessment that helps diagnose and direct therapy of the patient initially presenting as hemodynamically stable, but later deteriorating after admission to the emergency room [6]. Expanding on the value of a single venous lactate measurement, investigators have evaluated serial lactate concentration dynamics and found association between reduction in serial lactate concentration over time and improved outcomes [7–9]. This association may be related to roles of blood lactate as a volumetric integrator of cell glycolysis in hypoxic tissues, and the multiple roles of lactate as a substrate for cell metabolism and signaling [10, 11]. Signaling targets may be recipient cells in the same organ or in different organs such as the brain or heart [10, 11]. This concept of lactate as a signaling molecule has led to new studies on lactate in different disciplines including exercise physiology, critical care medicine, and oncology [12]. Currently, lactate is measured by intermittent blood samples with no clear guidelines on how often a measurement should be taken to give insight on real time physiological conditions. Controlling blood lactate using a strategy that includes a continuous lactate monitoring system has potential to improve treatment of critically ill or injured patients.

The goal of this study was to evaluate accuracy of a novel enzymatic, chemi-luminescence, optode-based Continuous Lactate Monitor (CLM). CLM measurements of subcutaneous tissue lactate concentration were compared to venous blood plasma lactate concentration during incremental cycling exercise challenges as a model for hyperlactatemia. We performed a bioequivalence analysis to test if subcutaneous lactate concentrations as measured by the CLM accurately reflect corresponding plasma lactate concentrations.

## Methods

### Participants

Six healthy individuals 18–45 y/o (4 males, 2 females) participated in the study (Table 1). Participants were active in moderate to intense exercise 3–5 times per week for at least 30 min each session. The Institutional Review Board at the University of California Irvine approved the study and written informed consent was obtained from all participants upon enrollment.

**Table 1:** Anthropometric characteristics and exercise data (n = 6)

Age (years)	32.0 ± 9.8
Gender (M/F)	4/2
Height (m)	1.71 ± 0.13
Body mass (kg)	75.9 ± 14.5
BMI (kg/m^2^)	25.8 ± 3.1
Exercise time (min)	14.9 ± 3.7
Bouts (quantity)	5 ± 1
Watt max	262.5 ± 81.7
HR max(bpm)	184 ± 8
RPE max (6–20)	19 ± 1

Values are means ± SD

*BMI* body mass index, *HR* heart rate, *RPE* rating of perceived exertion

### Study design

All participants performed an incremental exercise test with the CLM device in the Human Performance Laboratory at the University of California Irvine. Blood samples were taken and analyzed for lactate concentration before and during exercise, and during the subsequent rest period. The CLM was removed at the end of the rest period. The skin insertion site was reviewed by the study physician 48 h post exercise for signs of side effects.

Vital signs and anthropometric measurements were obtained before the exercise test. A urine pregnancy test for women subjects was performed to assess the exclusion criterion of pregnancy. During the exercise portion of the study, heart rate and rate of perceived exertion (RPE) were monitored. Prior to the initiation of the study an instruction was given to each participant, stating they can stop the test at any time for any reason. A physician was available to respond to and treat any medical issues encountered during the course of the study.

### Continuous lactate monitor insertion

**Fig. 1:**
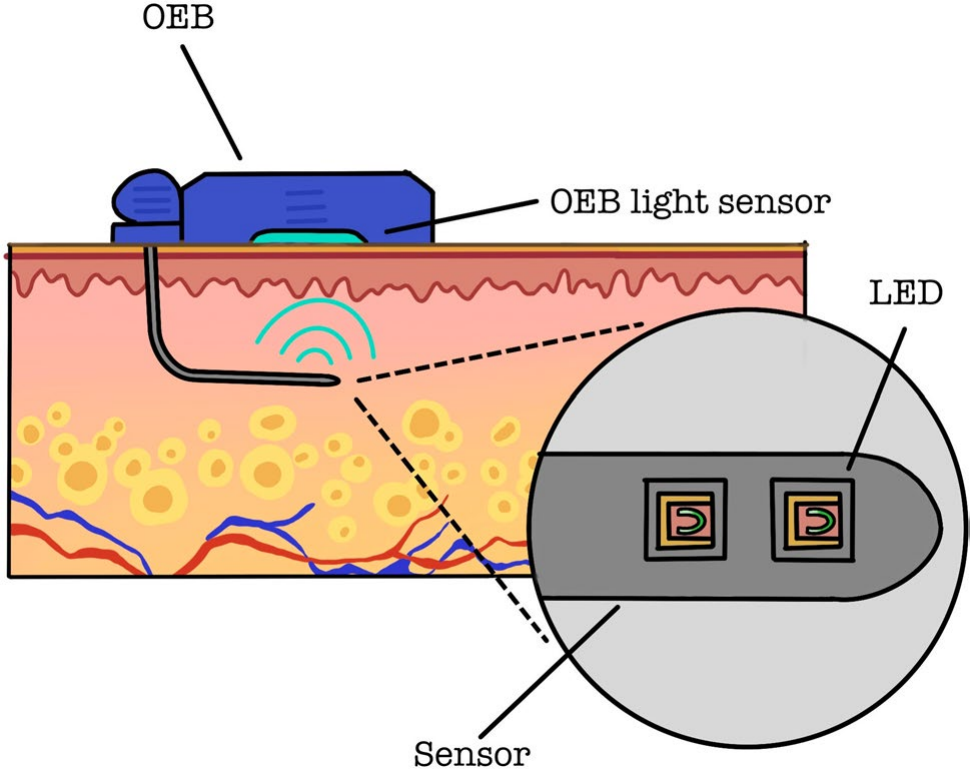
Continuous Lactate Monitor (CLM). CLM components include a flexible sensor with two LEDs at the distal tip. The sensor is inserted subcutaneously through an 18-gauge cannula. An opto-electronic backend (OEB) houses a photodetector (OEB light sensor) and receives light from the inserted sensor

The CLM components are shown in Fig. 1. A flexible sensor is inserted into the subcutaneous tissue. The tip of the sensor houses two light emitting diodes (LEDs). LED emission excites a lactate oxidase-coated chemi-luminescent dye, whose phosphorescence is quenched in proportion to local lactate concentration. CLM sensors were placed transcutaneously into the subcutaneous tissue of the postero-lateral trunk below the renal angle through the cannula of a standard 18-gauge catheter (Exel Safelet Catheter, Exelint International, Redondo Beach CA). The catheter-based sensor insertions were performed by a medical professional after a topical numbing cream (LMX-4 Topical Anesthetic Cream, Ferndale Laboratories Inc., Ferndale MI) was applied to the insertion site for a period of 45 min. Each CLM was activated prior to insertion to allow for visual confirmation of LED illumination. Following insertion, each sensor was connected to a custom Opto-Electronic Backend (OEB, Fig. 1) which was adhered to the skin with tape (Tegaderm Film, 3 M Health Care, St. Paul MN). The OEB was wired to a data acquisition system to control LED emission and digitize OEB light sensor signals.

#### Incremental exercise test

Each exercise challenge comprised a 3 to 12 min warm up period and up to seven, 4 min incremental workload bouts separated by rest intervals on a cycle ergometer (SensorMedics Ergoline 800S, Yorba Linda, CA). Cycle workload was increased by 25–100 watts every bout as prescribed by the exercise physiologist in order to achieve hyperlactatemia for each subject. Within the last 30 s of each bout, rate of perceived exertion (RPE) and heart rate (HR) were recorded, followed by a rest interval between each exercise bout to allow blood draw. After the exercise test was completed, participants rested on a reclining chair with continued monitoring and were offered water and food. The CLM was removed at the end of the final rest period.

#### Blood sampling and analysis

A registered nurse placed an IV cannula in the antecubital vein of the subject’s arm prior to initiation of exercise. Blood was drawn from the antecubital vein at up to 25 time points. Blood draws were performed before exercise, during exercise, and up to 120 min post exercise. Blood plasma lactate concentration was assayed from whole blood immediately following each blood draw using an iSTAT (Abbott, Abbott Park IL) and an Abbott CG4 + cartridge, used per manufacturer instructions.

#### Follow-up Visit

The study physician examined the insertion site of each subject 48 h post-study. The physician interviewed each subject to determine if they were experiencing any discomfort or pain and a physical examination of the CLM insertion site was performed. The physical examination involved assessment for infection, irritation, swelling, hematoma, purulent discharge, or other complications to the insertion site.

### Statistical analysis

CLM data was retrospectively calibrated to plasma lactate values using linear least squares regression following a time shift to account for dynamic delay. Dynamic delay was determined as the time lag that maximizes cross-correlation between linearly interpolated blood and CLM lactate timeseries [13, 14]:

1 $$\phi \left( \tau \right) = \sum\nolimits_{k = 0}^{N - 1} {x(k)y(k + \tau )}$$

where $\phi \left(\tau \right)$ is the cross-correlation as a function of time lag $\tau$.

Bioequivalence was assessed between each investigational CLM data and corresponding venous blood plasma lactate concentrations. Bioequivalence was determined from the area under the concentration–time curve ratio (AUCR) as defined in Eq. 2 in accordance with FDA guidance documentation [15]. Plasma lactate values were linearly interpolated to match CLM timepoints and the integrals of each waveform were calculated using the trapezoid rule [13, 14]. The AUCR was calculated as:

2 $$AUCR = \sum\nolimits_{k = 0}^{N - 1} {\frac{{y\left( {k - 1} \right) + y\left( k \right)}}{{x\left( {k - 1} \right) + x\left( k \right)}}}$$

where $x\left(k\right)$ and $y\left(k\right)$ are plasma lactate and CLM lactate timeseries respectively, $k$ is the time index, and $N$ is the timeseries length. The hypothesis is expressed as H_A_: 0.8 ≤ AUCR ≤ 1.25, where AUCR = 1 corresponds to exact bioequivalence. The lower and upper limits selected are commonly used in bioequivalence studies [15]. The corresponding null hypothesis is therefore H_0_: AUCR < 0.8 or AUCR > 1.25.

## Results

**Fig. 2:**
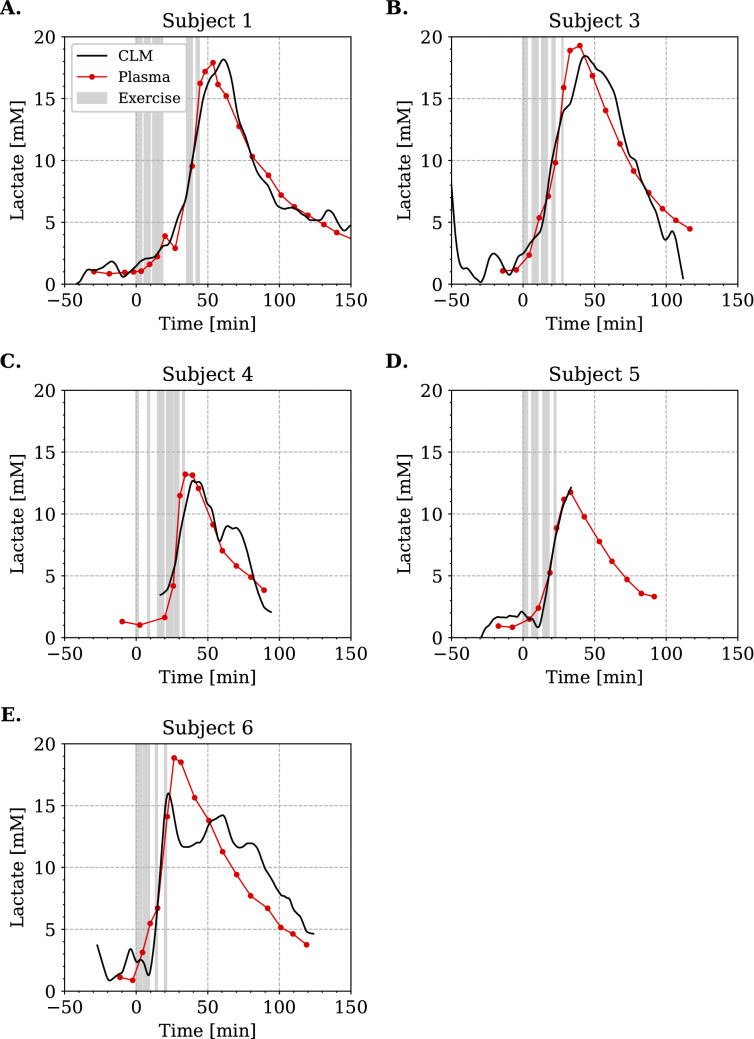
Lactate changes due to exercise. Calibrated CLM (black) and plasma lactate (red) concentration data. Gray columns indicate exercise bouts

CLM sensors were placed on six subjects. In one subject, subject 002, the CLM lost signal at the start of the exercise and no CLM data was collected. Of the 5 subjects where CLM data were available, 4 of the subject’s CLM maintained signal throughout the exercise and rest periods. In subject 005, the CLM maintained signal through the exercise portion of the study but lost signal after the exercise period. CLM sensor output was calibrated to blood plasma lactate concentrations via least squares regression of the two timeseries after alignment by cross correlation, as described above. AUCR and dynamic delay analyses were performed on all time periods when CLM data and plasma lactate data were both available. Plasma lactate measurements followed the expected trends: concentration of plasma lactate prior to exercise was relatively constant having low baseline values, lactate concentration during and immediately after exercise increased sharply, and lactate concentration during the rest period decreased. CLM data showed a similar trend. Calibrated CLM and iSTAT plasma lactate measurements for each subject are shown in Fig. 2 with exercise bouts indicated by gray columns. AUCR and dynamic delay were calculated for each working CLM, as reported in Table 2. The average AUCR across the 5 CLM was 1.025 supporting the bioequivalence hypothesis (H_A_). The average dynamic delay across all sensors was 5:13 (minutes: seconds).

**Table 2:** AUCR and dynamic delay

Subject	AUCR	Dynamic delay (min: sec)
001	0.990	5:45
003	1.022	10:21
004	1.057	3:27
005	0.997	3.39
006	1.058	2:52
Mean (± SD)	1.025 ± 0.032	5:13 ± 3:04

The study physician examined each sensor insertion site 48 h following device removal. No signs of infection were found in any subject and no subjects required further medical attention. Signs of erythema and edema were either not present or were classified as slight and not well-defined and did not require medical treatment.

## Discussion

Hyperlactatemia is a hallmark characteristic of shock state and the degree of increase in lactate concentration is directly related to the severity of the shock state and to mortality rate [17, 18]. Serial lactate measurements are recommended to monitor and assess patients in shock and to guide treatment [19]. Notably, it is the duration of hyperlactatemia that has been shown to be the best discriminant of survival and organ failure in septic shock [17]. A time course of lactate concentration can not only track the duration of hyperlactatemia, but can also track the accumulation and clearance of lactate from the blood. The area under the curve (AUC) of lactate timeseries provides an estimate of such accumulation. A continuous lactate monitor would be capable of providing real-time AUC calculation along with current lactate concentration and trends while reducing burden on treating staff through automated measurement. This real-time information may be further useful for risk stratification and guidance of therapy.

This study investigates bioequivalence between a novel subcutaneous continuous lactate monitor and blood plasma during high intensity interval exercise. Here, bioequivalence was assessed by AUCR analysis [15]. Mean AUCR across the five CLM devices (one per subject) was 1.025, corresponding to a less-than 10% difference in AUC between the CLM and blood plasma. We also observed a dynamic delay in CLM values relative to the blood plasma. The average delay time was 5:13 (minutes: seconds) and is similar to dynamic delays of other subcutaneous continuous monitors such as commercial continuous glucose monitors [16]. This moderate delay is not prohibitive to use considering the typical rate of change of lactate in conditions such as shock is on the order of 10–20% change per 6 h [20, 21], much lower than the rate of lactate change seen in our high intensity interval exercise study. It is reasonable to assume that differences between interstitial (measured by our sensor) and blood plasma lactate concentrations will increase during hypoperfusion, a condition typical to states of shock, including septic shock. But surprisingly, Kopterides et al. found the opposite effect. They used microdialysis to frequently measure lactate concentration in subcutaneous adipose tissue within a population of patients including 96 diagnosed with septic shock [22]. The authors found that during shock, tissue lactate is better correlated with blood lactate as compared to non-shock states. Furthermore, changes in tissue lactate concentration preceded changes in blood lactate changes in shock states. This observation suggests that our CLM could be used to indicate clinical action at a time point earlier than would be indicated by intermittent blood analysis.

The AUCR metric used in our bioequivalence analysis does not report on waveform shape. However, in our study it was observed that for two of the subjects the shape of the CLM curve differed from that of the plasma lactate following exercise. The CLM signals from subjects 4 and 6 exhibited a drop in lactate after the exercise session, followed by an increase in lactate, a trend not observed in the blood plasma. This difference in trends may be due to physiological factors such as changes in blood flow, lactate diffusion rate, and oxygen concentration at the CLM site. More data is needed to fully understand the observation. Subject-to-subject differences in AUCR and dynamic delay time may be related to individual subject physiology including exercise recovery or differences in the wound response at the site of the sensor. Other sources of variance could include sensor-to-sensor variation, subject posture during the rest period, and sensor insertion. In fact, we attribute the CLM failure in subject 2 and the loss of CLM signal post-exercise in subject 4 to problematic insertion. Specifically, we observed axial displacement or rotation of the CLM sensor around its long axis, resulting in misalignment of the CLM optode and OEB unit and loss in signal. When the insertion did not fail, the CLM was shown to achieve bioequivalence with the plasma lactate.

We are currently working to improve the insertion system and to further miniaturize the sensor while achieving consistency-in-manufacturing in order to provide a sensor with reliable calibration and stable operation. We are also extending the capabilities our CLM to measure multiple analytes on a single inserted probe. With additional LEDs, each coated with a specific reagent, the CLM is currently measuring glucose, lactate, and pO_2_. This CLM embodiment is being tested clinically in the context of glycemic control during exercise in subjects with type 1 diabetes. Efforts are underway to include measurements of pH, pCO_2_, and peptide hormones, along with other metabolic and hemodynamic markers, where all assays are integrated into a single flexible sensor.
